# Excess Folic Acid Increases Lipid Storage, Weight Gain, and Adipose Tissue Inflammation in High Fat Diet-Fed Rats

**DOI:** 10.3390/nu8100594

**Published:** 2016-09-23

**Authors:** Karen B. Kelly, John P. Kennelly, Marta Ordonez, Randal Nelson, Kelly Leonard, Sally Stabler, Antonio Gomez-Muñoz, Catherine J. Field, René L. Jacobs

**Affiliations:** 1 Department of Agricultural, Food & Nutritional Science, University of Alberta, Edmonton, AB T6G2P5, Canada; kkelly1@ualberta.ca (K.B.K.); jkennell@ualberta.ca (J.P.K.); rn1@ualberta.ca (R.N.); kmd4@ualberta.ca (K.L.); cjfield@ualberta.ca (C.J.F.); 2 Department of Biochemistry and Molecular Biology, Faculty of Science and Technology, University of the Basque Country (UPV/EHU), Bilbao 48080, Spain; marta.ordonez87@gmail.com (M.O.); antonio.gomez@ehu.es (A.G.-M.); 3 Department of Medicine, University of Colorado School of Medicine, Aurora, CO 80206, USA; Sally.Stabler@ucdenver.edu; 4 Department of Biochemistry, University of Alberta, Edmonton, AB T6G2R7, Canada

**Keywords:** folic acid, obesity, metabolic syndrome, adipose tissue

## Abstract

Folic acid intake has increased to high levels in many countries, raising concerns about possible adverse effects, including disturbances to energy and lipid metabolism. Our aim was to investigate the effects of excess folic acid (EFA) intake compared to adequate folic acid (AFA) intake on metabolic health in a rodent model. We conducted these investigations in the setting of either a 15% energy low fat (LF) diet or 60% energy high fat (HF) diet. There was no difference in weight gain, fat mass, or glucose tolerance in EFA-fed rats compared to AFA-fed rats when they were fed a LF diet. However, rats fed EFA in combination with a HF diet had significantly greater weight gain and fat mass compared to rats fed AFA (*p* < 0.05). Gene expression analysis showed increased mRNA levels of peroxisome proliferator-activated receptor γ (PPARγ) and some of its target genes in adipose tissue of high fat-excess folic acid (HF-EFA) fed rats. Inflammation was increased in HF-EFA fed rats, associated with impaired glucose tolerance compared to high fat-adequate folic acid (HF-AFA) fed rats (*p* < 0.05). In addition, folic acid induced PPARγ expression and triglyceride accumulation in 3T3-L1 cells. Our results suggest that excess folic acid may exacerbate weight gain, fat accumulation, and inflammation caused by consumption of a HF diet.

## 1. Introduction

The metabolic syndrome, which encompasses excess abdominal adiposity, insulin resistance, dyslipidaemia, and hypertension, represents the largest public health challenge in developed countries [1]. The rise in metabolic syndrome prevalence in recent decades has been mirrored by changes in dietary patterns, reflecting increased nutrient availability [2]. Diets rich in fat and rapidly-digestible carbohydrates have increased total energy intake [2]. At the same time, fortification of staple foods and widespread supplement use has increased folic acid intake in many Western countries, placing importance on investigations into possible adverse effects [3].

Folates are a family of structurally-similar compounds involved in the transfer of one-carbon units for the production of nucleotides used in DNA synthesis; for the methylation of a variety of biological substrates; and for cell division [4]. These functions make folates especially important during the anabolic stages of foetal and childhood development [4]. Sources of natural folates (pteroylpolyglutamates) include green leafy vegetables, orange juice and legumes [4]. Folic acid (monoglutamate) is a synthetic member of the folate family commonly used in fortified foods and supplements due to its stability and low cost [4]. The current Recommended Daily Allowance (RDA) for folate is 400 μg Dietary Folate Equivalents (DFEs)/day for the general adult population [5]. While mandatory folic acid fortification of grains since 1998 has reduced the incidence of neural tube defects (NTDs) and other developmental disorders in Canada and the USA, population-wide intake of folic acid has increased to unprecedented levels, leading to concern that there may be adverse consequences [3,6,7]. Children and elderly populations are likely to have high folic acid intake because large proportions of their diet typically consist of cereals and bread [3]; while pregnant women are likely to have high intakes due to high supplement use [8]. High folic acid intake by women planning pregnancy is prevalent in many countries, including countries without mandatory folic acid fortification, due to worldwide recommendations for this population to consume 400 µg/day folic acid for the prevention of neural tube defects [9,10]. Human observational evidence has linked high folic acid intake to increased risk of colorectal and prostate cancer [11,12]; impaired immune function [13,14], and impaired cognition [15]. Further observational evidence has linked folate status to obesity, sparking investigations into the relationship between folic acid intake and lipid and energy metabolism [16,17].

Maternal excess folate [17,18] or methyl donor [19] intake during pregnancy in animal models causes weight gain or components of the metabolic syndrome in offspring. These effects may be more pronounced when offspring are fed a high fat diet [20,21]. In humans, high erythrocyte folate status during pregnancy was associated with increased fat mass of children at six years of age [22]. Folic acid appears to influence energy and lipid metabolism by modulating DNA methylation and gene expression patterns [17,18,23]. Diet-gene interactions remain important determinants of health throughout the lifespan [24], and so excess folic acid may continue to promote changes to energy and lipid metabolism in adulthood. However, the effects of excess folic acid intake on metabolic syndrome risk and adiposity in adulthood remains poorly understood.

The effects of excess dietary folic acid intake on the liver, an important site of both folate and lipid metabolism, have been investigated in rodent models [23,25]. Excess folic acid intake may promote changes to one carbon metabolic pathways and gene expression patterns, leading to liver injury [25]. There is evidence that the influence of methyl donors, including folic acid, on gene expression may be tissue-, site-, and gene-specific, and so investigations into the influence of excess folic acid on other tissues (e.g., adipose) are warranted [3].

The aim of our study was to investigate the effects of excess folic acid (EFA) intake compared to adequate folic acid (AFA) intake on metabolic health of rats. We hypothesized that consumption of a diet containing EFA would induce changes to lipid and glucose metabolism. High fat diets are commonly used to study weight gain and components of the metabolic syndrome in animal models. Therefore, we conducted our investigations in the setting of a 15% of energy low fat (LF) and a 60% of energy high fat (HF) diet. Our data suggest that EFA, in combination with a HF diet increases weight gain, adipose tissue mass and markers of inflammation compared to AFA, and that these effects are not seen in the setting of a LF diet. We conducted supporting experiments in vitro, the results of which suggest that folic acid can increase triglyceride accumulation in 3T3-L1 cells by inducing peroxisome proliferator-activated receptor γ (PPARγ).

## 2. Materials and Methods

### 2.1. Animals and Diets

All procedures were approved by the University of Alberta’s Institutional Animal Care Committee (AUP00000175) in accordance with guidelines of the Canadian Council on Animal Care. All animals had free access to food and water and were housed on a 12-h light-dark cycle. The diet was supplied by Harlan Teklad, with basal diet formulations designed to AIN-93G specifications. In the first feeding trial, twelve eight-week old male Sprague–Dawley rats were fed a 15% of energy low fat diet with excess folic acid (7.5 mg/kg diet) or control levels of folic acid (0.75 mg/kg diet) (Table 1) [23]. In the second feeding trial, twelve rats were fed a 60% of energy high fat diet containing excess folic acid (7.5 mg/kg) or control levels of folic acid (0.75 mg/kg) (Table 1). Food intake and body weights were recorded three times weekly for the duration of the experiments. Food intake is reported as the average daily intake over the 12-week feeding period. During the eighth weeks of feeding, body composition was analyzed by magnetic resonance imaging (MRI). Animals were euthanized after 12 weeks on diet. Fasting blood was collected in ethylenediaminetetraacetic acid (EDTA)-coated vials by cardiac puncture and plasma was collected after centrifugation of blood at 3000× *g* for 10 min. Tissues were weighed and snap frozen in liquid nitrogen before being stored at −80 °C until analysis.

### 2.2. Histological Analysis of Adipose and Liver Samples

Adipose tissue was collected, dehydrated, and embedded in paraffin. Cross-sections of tissue (5 μm) were prepared and stained with haematoxylin and eosin (H and E). Adipocyte size was estimated using ImageJ software, (the US National Institutes of Health, Bethesda, MD, USA).

### 2.3. Glucose Tolerance Tests

Eight weeks after initiation of the high fat diet (HFD) feeding trial, rats were fasted overnight before receiving 2 g/kg glucose by intraperitoneal (IP) injection. Blood samples were collected by tail vein bleeding at 15, 30, 60, 90, and 120 min.

### 2.4. Plasma Measurements

Plasma glucose and alanine aminotransferase (ALT) were measured using commercially available kits (WAKO Diagnostics (Mountain View, CA, USA) and Biotron (Diagnostic Inc., Hemet, CA, USA), respectively). Plasma insulin was measured by ELISA (ALPCO, Salem, NH, USA). Plasma levels of metabolites in the one carbon cycle, including folate, total homocysteine, methionine, cysteine, *N*,*N*-dimethylglycine, *N*-methylglycine, glycine, serine, cystathionine, α-aminobutyrate, were measured by capillary stable isotope dilution gas chromatography/mass spectrometry, as previously described [26]. Neutral lipids in plasma were quantified by gas-liquid chromatography as described previously [27], with tridecanoin as an internal standard.

### 2.5. Culture of 3T3L1 Adipocytes

3T3-L1 cells were cultured to confluence in Dulbecco’s modified Eagle’s medium (DMEM) supplemented with 10% (*v*/*v*) fetal bovine serum (FBS). At two days post-confluence (designated day 0), cells were induced to differentiate with DMEM supplemented with 10% (*v*/*v*) FBS, 1 μM dexamethasone, 0.5 mM isobutylmethylxanthine, 1 μg/mL insulin. Cells were incubated with 9 µM (standard level) or 20 µM (supplemented) folic acid. Differentiation media was refreshed daily. After 48 h, the media were replaced with DMEM supplemented with 10% FBS and 1 μg/mL insulin and the same level of folic acid that was used during differentiation. The cell media was refreshed every 24 following differentiation.

### 2.6. Analytical Procedures

Tissue levels of cytokines and chemokines were quantified using ELISA kits, according to manufacturer’s instructions (Preprotech, Rocky Hill, NJ, USA or eBioscience, San Diego, CA, USA). To measure triglycerides in 3T3L1 adipocytes, cells were rinsed three times in sterile PBS then collected in 2 mL PBS by scraping. Cells were disrupted by vortexing, followed by sonication 3 × 15 s. Triglycerides were measured by colorimetric assay, according to the manufacturer’s instructions (Sekisui Diagnostics, Lexington, MA, USA).

### 2.7. mRNA Quantification

mRNA was quantified as previously described [28]. Briefly, total RNA was isolated from tissue using Trizol reagent (Invitrogen, Carlsbad, CA, USA). RNA was then reverse transcribed using Superscript II (Invitrogen, Carlsbad, CA, USA). The Universal Probe Library (Roche Diagnostics, Indianapolis, IN, USA) was used to design primers and corresponding probes for each gene being evaluated. Quantitative PCR was run in triplicate on the Biomark system (Fluidigm, South San Francisco, CA, USA) for 40 cycles. Relative mRNA expression for each gene was calculated using the ΔΔCT method, normalized to cyclophilin.

### 2.8. Statistical Analysis

Data are expressed as the means ± standard error of the mean (SEM). Data were analyzed using one-way ANOVA or Student’s *t*-test where appropriate. All analyses were done using GraphPad Prism software version 6.00 for Windows (GraphPad Software, La Jolla, CA, USA). A *p*-value < 0.05 was taken as statistically significant.

## 3. Results

### 3.1. Excess Folic Acid Intake Does Not Influence Body Weight, Body Composition, or Glucose Tolerance on a Low Fat Diet

**Figure 1:**
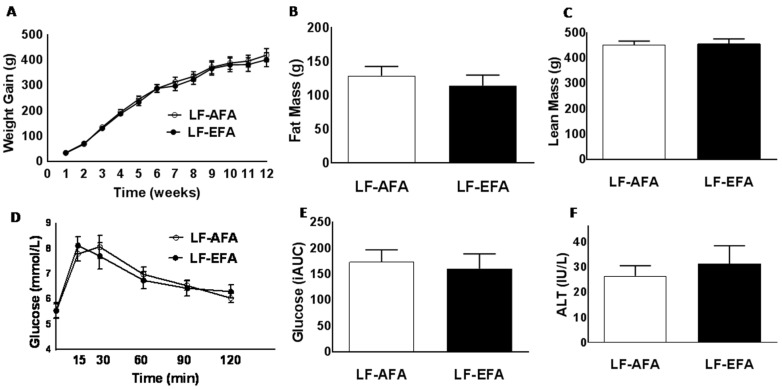
Excess folic acid intake does not influence body weight, body composition or glucose tolerance on a low fat diet (**A**); growth curves (**B**); fat mass (**C**); lean mass (**D**); glucose tolerance (**E**); area under the glucose curve, and (**F**) plasma ALT, of rats fed 15% LF diet with excess or adequate folic acid. Values are means ± SEM, * *p* < 0.05.

Rats were fed a LF diet with either AFA or EFA for 12 weeks. LF-AFA and LF-EFA fed rats had similar weight gain, fat mass and lean mass over the 12 weeks study period (Figure 1A–C). Glucose tolerance tests showed no difference in rate of glucose clearance between LF-AFA and LF-EFA fed rats (Figure 1D,E). Plasma ALT concentration, a marker of liver injury, was not different between groups (Figure 1F).

### 3.2. Excess Folic Acid Intake Increases Weight Gain, Fat Mass and Glucose Intolerance on a High Fat Diet

We next investigated the influence of AFA or EFA in rats challenged with a diet containing 60% kilocalories from fat for 12 weeks. Plasma homocysteine concentrations were significantly lower in HF-EFA fed rats (3.28 ± 0.17 compared to 2.650 ± 0.14 μM/L) at the end of the study period (Table 2). Plasma methionine and glycine concentrations were also lower in HF-EFA fed rats, while plasma folate concentration was similar between groups (Table 2). There was no significant difference in plasma concentrations of triglycerides, cholesterol, or cholesterol ester in rats fed HF-AFA or HF-EFA diets (Table 2).

**Figure 2:**
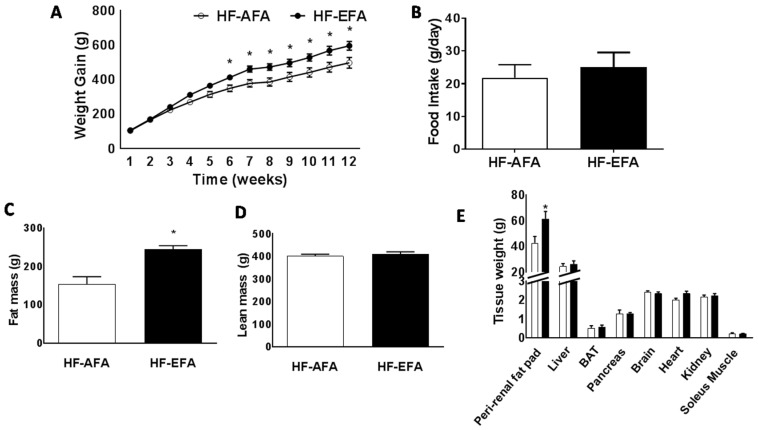
Excess folic acid intake increases weight gain and fat mass on a high fat diet. (**A**) Growth curves; (**B**) food intake; (**C**) fat mass; (**D**) lean mass; and (**E**) tissue weights. Values are means ± SEM, * *p* < 0.05.

**Figure 3:**
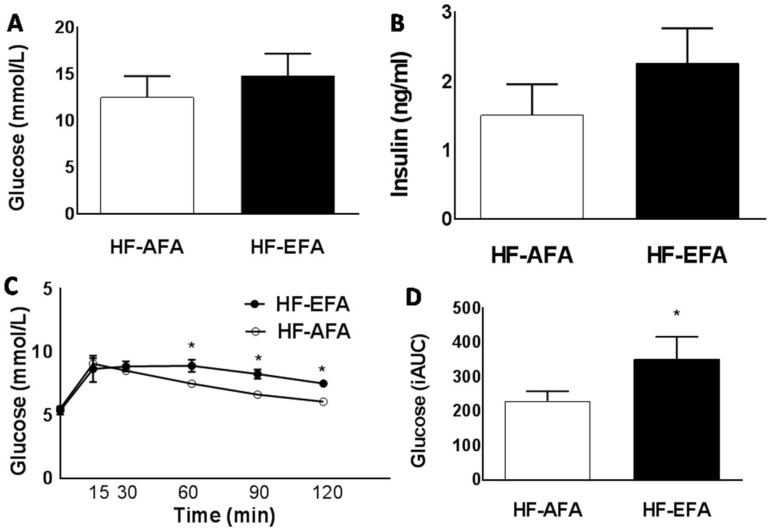
Excess folic acid intake impairs glucose tolerance on a high fat diet. (**A**) Fasting plasma glucose; (**B**) fasting plasma insulin; (**C**) blood glucose concentrations at different time points (15, 30, 60, 90, 120 min) after an intraperitoneal (IP) glucose injection; and (**D**) area under the glucose curve, for male rats fed 60% HF diet with excess or adequate folic acid. Values are means ± SEM, * *p* < 0.05.

HF-EFA fed rats had 14% greater weight gain compared to HF-AFA fed controls after 12 weeks (Figure 2A). Estimated daily food intake was similar between groups (Figure 2B). Fat mass accounted for this difference in weight, with HF-EFA fed rats developing larger peri-renal fat pads (Figure 2C,D). There was no difference in lean body mass between HF-EFA and HF-AFA fed rats (Figure 2E). Fasting plasma glucose and insulin levels were similar between HF-EFA and HF-AFA fed rats (Figure 3A,B). However, IP glucose tolerance tests showed that HF-EFA fed rats had impaired glucose clearance compared to HF-AFA fed rats, as indicated by a significantly greater area under the glucose curve (Figure 3C,D). Therefore, EFA intake exacerbates weight gain, fat mass, and glucose intolerance in rats fed a HF diet.

### 3.3. Excess Folic Acid Increases Adipose Tissue Size and Mass By Inducing Lipogenic Genes in High Fat Diet-Fed Rats

**Figure 4:**
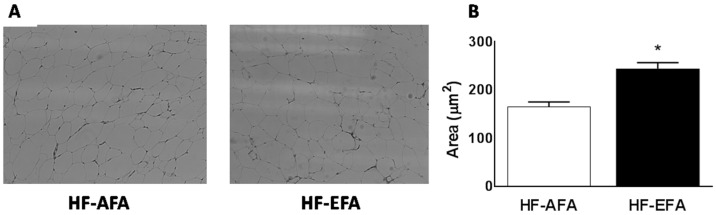
HF-EFA fed rats had larger adipocytes than HF-AFA fed rats. (**A**) Adipose tissue histology after H and E staining; (**B**) adipocyte size was quantified using ImageJ software. Values are means ± SEM,* *p* < 0.05.

**Figure 5:**
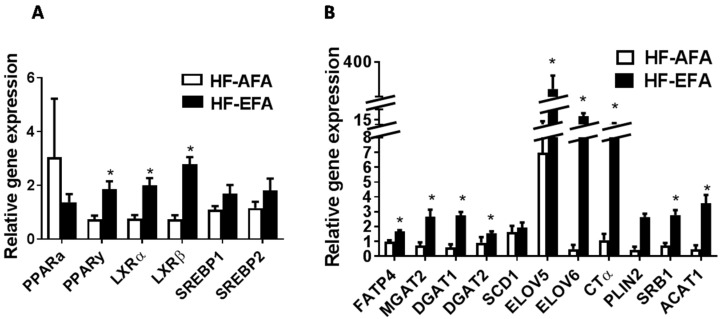
Gene expression analysis in adipose tissue of HF-EFA shows increased levels of lipogenic mediators. Relative mRNA levels of (**A**) transcription factors and (**B**) genes involved in lipid synthesis, storage and transport, in adipose tissue of HF-AFA and HF-EFA fed rats. Values are means ± SEM, * *p* < 0.05.

Histologic examination of visceral adipose tissue after hematoxylin and eosin (H and E) staining showed increased adipocyte size in HF-EFA fed rats compared to HF-AFA fed controls (Figure 4). To further investigate this increased adiposity, we measured expression of key transcriptional regulators of lipid metabolism (Pparg, Srebf1, Srebf2, Nr1h2, Nr1h3), and lipogenic genes in adipose tissue. PPARγ regulates genes involved in lipid uptake and storage. Adipose tissue PPARγ mRNA was 2.5-fold higher in HF-EFA fed rats compared to HF-AFA fed controls (Figure 5A). Liver X receptor (LXR)-α and -β (encoded by Nr1h3 and Nr1h2) are nuclear transcription factors that have roles in adipose tissue lipid metabolism as well as inflammation [29]. LXR-α and -β mRNA levels were significantly higher in HF-EFA fed rats compared to HF-AFA fed rats (Figure 5A). Furthermore, there was increased mRNA levels of triglyceride synthetic genes (MGAT1, DGAT1 and DGAT2); genes involved in elongation (ELOV5 and ELOV6); and markers of adipogenesis (PLIN2) in adipose tissue of HF-EFA fed rats compared to HF-AFA fed rats (Figure 5B). 

Increased adipocyte size and number increases demand for phosphatidylcholine (PC), which surrounds adipose tissue lipid droplets in a monolayer. Consistent with this increased demand, mRNA levels of the rate limiting enzyme in PC biosynthesis, cytidine triphosphate: phosphocholine cytidylyltransferase (CT) α, was increased in adipose tissue of HF-EFA fed rats compared to HF-AFA control rats (Figure 5B). Taken together, these observations indicate that EFA intake may induce lipogenic transcription factors and some of their dependent genes to promote adiposity in the setting of a HF diet. 

### 3.4. Excess Folic Acid Increases Inflammation in White Adipose Tissue

**Figure 6:**
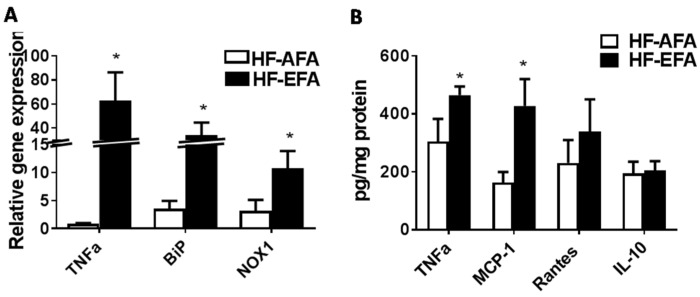
Inflammatory markers are increased in adipose tissue of HF-EFA compared to HF-AFA fed rats. (**A**) Relative mRNA levels of genes related to inflammation; and (**B**) protein levels of TNFα, MCP-1, Rantes, IL-10 in pg/mg protein, in adipose tissue of HF-EFA and HF-AFA fed rats. Values are means ± SEM, * *p* < 0.05.

White adipose tissue (WAT), in addition to its role in energy storage, secretes adipocytokines and chemokines which link increased fat mass to local and systemic insulin resistance [30]. In obesity, excess adipose tissue accumulation precedes immune cell infiltration and production of pro-inflammatory cytokines [30]. We measured adipose tissue protein levels of the chemokines monocyte chemoattractant protein-1 (MCP-1) and regulated on activation, normal T cell expressed and secreted (RANTES), and the cytokines tumor necrosis factor α(TNFα) and interleukin 10 (IL-10), as markers of inflammatory status. MCP-1 is a chemokine involved in monocyte and macrophage recruitment to adipose tissue [31]. Levels of MCP-1 were significantly higher in adipose tissue of HF-EFA fed rats (Figure 6B). Chronic low-grade inflammation after macrophage recruitment to adipose tissue can influence local and systemic insulin sensitivity. Protein and mRNA levels of TNFα, an inflammatory cytokine secreted by macrophages, were significantly higher in adipose tissue of HF-EFA fed rats compared to HF-AFA fed controls (Figure 6A,B). Adipose tissue levels of the cytokine IL-10 were not different between HF-AFA and HF-EFA fed rats (Figure 6B). Transcript levels of the inflammatory markers NADPH oxidase 1 (NOX1) and binding immunoglobulin protein (BiP) were found to be significantly increased in adipose tissue of HF-EFA fed rats after 12 weeks on diet (Figure 6A).

### 3.5. Excess Folic Acid Promotes Triglyceride Accumulation in Mature 3T3L1 Adipocytes

**Figure 7:**
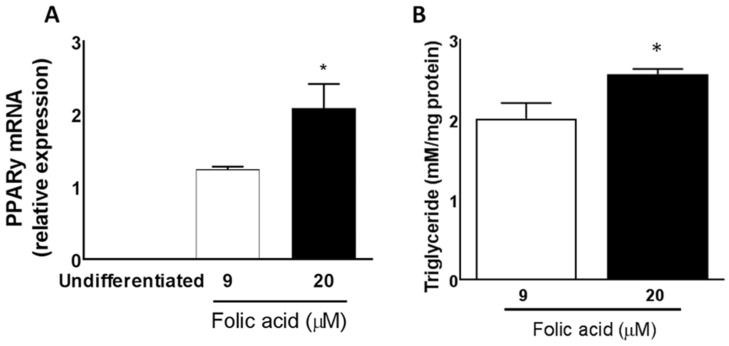
Folic acid increases PPARγ and triglyceride levels in a dose-dependent manner in cultured 3T3-L1 cells. (**A**) Relative PPARγ mRNA levels in cells cultured with 9 µM or 20 µM folic acid and (**B**) Triglyceride levels in cells after treatment with 9 µM compared to 20 µM folic acid. Values are means ± SEM, * *p* < 0.05.

We used 3T3-L1 cells to assess the lipogenic capacity of folic acid in vitro (Figure 7). We measured PPARγ mRNA expression as a marker of adipocyte differentiation in undifferentiated and mature 3T3-L1 cells cultured with 9 µM (normal) and 20 µM folic acid (excess). PPARγ was undetectable in undifferentiated 3T3-L1 cells. Treatment of differentiated adipocytes with 9 µM folic acid increased PPARγ expression and triglyceride (TG) accumulation compared to undifferentiated cells. High folic acid (20 µM) further promoted PPARγ expression and TG accumulation, indicating a dose-response to folic acid treatment in 3T3-L1 cells. These results support those found in adipose tissue of rats fed HF-EFA compared to HF-AFA diets.

## 4. Discussion

Our results show that excess dietary folic acid exacerbates fat mass gain, adipose tissue inflammation, and systemic glucose intolerance in rats fed a HFD. These metabolic complications were not observed in rats fed a LF-EFA diet. Energy dense diets have long been implicated in fat mass gain and metabolic syndrome development [2]. Our results suggest that high dietary folic acid may aggravate these effects.

The body weight of rats in the HF-EFA group after 12 weeks was 14% higher than in rats in the HF-AFA group, with increased fat mass accounting for this difference in weight. Histological examination of adipose tissue revealed that HF-EFA fed rats had larger adipocytes. The capacity of folic acid or methyl-rich diets to promote weight gain has been reported previously in young rats fed 5 mg/kg folic acid [23], as well as in maternal rats fed a high fat diet enriched in methyl-containing vitamins [32]. Our data supports these findings and suggests that a high folic acid diet promotes fat gain.

Adipogenesis is a multistep process which is controlled by transcription factors that promote adipocyte development and adipose tissue expansion [33]. PPARγ is an important regulator of adipogenesis and is sensitive to nutrient composition of the diet [34]. Most pro-adipogenic factors appear to function by stimulating PPARγ [34]. Gene expression analysis showed that PPARγ mRNA levels were approximately three times higher in adipose tissue of HF-EFA fed rats compared to HF-AFA fed controls. Folic acid supplementation has previously been shown to decrease PPARγ promoter methylation in rat liver leading to an increase in PPARγ gene expression [35]. Other studies have reported that methylation status influences expression of key genes involved in lipid metabolism [23,36]. Therefore, it is conceivable that the increase in PPARy expression observed in our study is related to changes to methylation status of adipose tissue PPARy, induced by excess folic acid. Addition of folic acid to the medium stimulated PPARγ expression and increased TG storage in 3T3-L1 cells, which further supports the ability of folic acid to stimulate adipogenesis. The capacity of folic acid to stimulate PPARγ expression in adipose tissue has been previously reported [23]. LXRα and LXRβ, transcriptional regulators of lipid and glucose metabolism [29], mRNA levels were increased in HF-EFA compared to HF-AFA fed rats. These data suggest that the increased adiposity observed in HF-EFA fed rats is a result of increased induction of lipogenic transcription factors and their target genes in adipose tissue.

Excessive adiposity is associated with metabolic stress and inflammation which is induced when macrophages migrate to adipose tissue and secrete pro-inflammatory cytokines. HF-EFA fed rats had higher adipose tissue protein levels of MCP-1, a chemokine that recruits macrophages to obese adipose tissue [37]. Consistent with this observation, protein and mRNA levels of the pro-inflammatory cytokine TNFα were significantly elevated in adipose tissue of HF-EFA fed rats compared to HF-AFA fed controls. Elevated TNFα is linked to insulin resistance in humans and animals. Consistent with significantly increased TNFα protein and mRNA levels in adipose tissue, HF-EFA fed rats had impaired glucose tolerance compared to HF-AFA fed rats. Maternal EFA supplementation has previously been shown to impair glucose metabolism in HF diet fed offspring [20]. Our results support this cross-generational evidence and demonstrate a similar effect in a single-generational study of rats. Thus, our data suggest that the mechanism linking EFA intake to systemic glucose intolerance may be TNFα- mediated inflammation in adipose tissue.

Metabolic complications in adipose tissue generally precede similar complications in other tissues, such as the liver [38,39]. TNFα produced in adipose tissue may migrate into the circulation and exert pro-inflammatory effects on other tissues [38,39]. In addition, when adipose tissue has reached its capacity to store fatty acids there may be a ‘spill-over’ of fat to other tissues. This amplifies the importance of identifying and controlling factors that influence inflammation and glucose intolerance in adipose tissue as they can ultimately compromise systemic health. After observing increased triglyceride accumulation and inflammation in adipose tissue of HF-EFA fed rats, we examined hepatic neutral lipid accumulation and mRNA levels of inflammatory genes. No significant difference in triglyceride accumulation was observed, and mRNA levels of a select number of inflammatory genes were not increased in the HF-EFA fed groups (data not shown). It has been previously reported that while HFD-induced inflammation can occur relatively quickly in adipose tissue of murine models, hepatic inflammation can take up to 40 weeks to develop, suggesting that our 12 weeks study period may have been too short to observe effects of folic acid in the liver [39]. Twenty-four weeks of supplementary folic acid (20 mg/kg) was sufficient to alter lipid metabolism and cause liver injury in mildly MTHFR-deficient rats [25].

While there is increasing evidence that excess dietary folate intake may have adverse effects, it is important to acknowledge the necessity of adequate levels of folate for good health. The role of dietary folate in NTD prevention is widely acknowledged, but adequate folate is also essential for regulating lipid metabolism, especially in the liver where most methylation reactions occur [40]. Folate deficiency can lead to liver damage and steatosis, with impaired phosphatidylcholine (PC) synthesis and increased expression of lipogenic genes cited as possible mechanisms [40]. Folate is also essential for re-methylation of homocysteine to methionine, which can be used to synthesize protein, or recycled to *S*-adenosylmethionine (*S*-AdoMet), a key methyl donor [4]. Excessively high folic acid intake can increase levels of unmetabolized folic acid in circulation [13], and its transport into tissues [41]. Some of the adverse health effects linked to excessive folic acid intake have been attributed to unmetabolized folic acid. However, the molecular mechanisms involved in processing unmetabolized folic acid within different tissue and cell types, and the biological effects of this, is poorly understood. It is conceivable that the increased adiposity observed in our study is a result of altered gene methylation and expression patterns in adipose tissue by folic acid.

We decided to supplement the rat diet with 10 times the adequate level of folic acid. While this could be considered superphysiological, we have previously reported that a sub-set of pregnant Alberta women consume over 4 mg/day (10 times the RDA for the general population) of folic acid [8]. Rats reduce folic acid to tetrahydrofolate at a rate that far exceeds that seen in humans [42]. Therefore, the observation that 7.5 mg/kg folic acid induced fat mass gain and inflammation in rats despite their relatively high dihydrofolate reductase activity is striking. Interestingly, Burdge et al. reported that folic acid supplementation (5 mg/kg) induced weight gain in juvenile rats fed a high fat diet [23]. Nonentheless, the inter-species differences in capacity to handle high doses of folic acid need to be considered when drawing conclusions from animal studies and places importance on establishing the metabolic effects of excess folic acid in human populations.

## 5. Conclusions

Folic acid fortification has succeeded in reducing incidence of neural tube defects in North America. However, there is increasing evidence that high folic acid intake may have consequences. Our findings suggest that in adult males the dual insult of a high fat diet combined with excess folic acid may promote fat mass gain, adipose tissue inflammation and systemic glucose intolerance. Obesity and the metabolic syndrome represent major public burdens and the link between excess folic acid and metabolic complications warrants further investigation given the ubiquitous presence of folic acid in the human food chain.

## Figures and Tables

**Table 1:** Composition of diets (per kilogram diet).

Ingredients	LF-AFA	LF-EFA	HF-AFA	HF-EFA
Folic acid (mg)	0.75	7.5	0.75	7.5
l-cysteine (g)	3	3	4	4
Corn starch (g)	263.7	263.7	-	-
Sucrose (g)	209.7	209.7	106.3	106.3
Maltodextrin (g)	130	130	160	160
Soybean Oil (g)	60	60	30	30
Lard (g)	-	-	310	310
Cellulose (g)	50	50	20	20
Pectin (g)	50	50	50	50
Succinylsulphathiazole (g)	10	10	10	10
Vitamin-free casein (g)	195	195	265	265
Mineral Mix, AIN-93G (g)	35	35	48	48
Tertiary-Butylhydroquinone (mg)	12	12	3400	3400
Choline bitartrate (g)	2.5	2.5	3	3
Niacin (mg)	30	30	63	63
Calcium pantothenate (mg)	16	16	34	34
Pyridoxine HCl (μg)	7	7	15	15
Thiamin HCl (μg)	6	6	13	13
Riboflavin (mg)	6	6	13	13
Biotin (μg)	200	200	400	400
Vitamin B12 (μg)	25	25	40	40
dl-alpha tocopheryl acetate (500 IU/g) (mg)	150	150	315	315
Vitamin A palmitate (500,000 IU/g) (mg)	8	8	17	17
Cholecalciferol (500,000 IU/g) (mg)	2	2	4	4
Phylloquinone (μg)	800	800	1600	1600

**Table 2:** HF-EFA fed rats experience alterations in plasma one carbon metabolite profile, while plasma lipids remain unchanged, compared to HF-AFA fed rats.

	HF-AFA	HF-EFA
**Plasma one carbon metabolites**		
Folate (nmol/L)	41.56 ± 0.50	41.30 ± 0.72
Homocysteine (μM)	3.28 ± 0.17	2.650 ± 0.14 *
Methionine (μM)	69.73 ± 2.93	62.58 ± 1.01 *
Dimethylglycine (μM)	13.10 ± 0.98	15.68 ± 0.95
Methylglycine (μM)	7.19 ± 0.48	6.21 ± 0.31
Glycine (μM)	425.0 ± 21.6	345.5 ± 24.0 *
Serine (μM)	339.2 ± 11.2	364.5 ± 10.9
Cystathionine (nM)	914.7 ± 69.2	763.3 ± 37.2
Cysteine (nM)	311.3 ± 4.5	305.7 ± 9.7
α-aminobutyrate (μM)	27.68 ± 2.6	35.60 ± 4.0
**Plasma lipids**		
Triglyceride (µg/mL)	292.6 ± 43.26	211.8 ±46.21
Cholesterol Ester (µg/mL)	72.92 ± 5.87	60.18 ± 7.97
Free Cholesterol (µg/mL)	134.4 ± 18.65	118.1 ± 15.41

Values are means ± SEM, * *p* < 0.05.
